# Is the fluid mosaic (and the accompanying raft hypothesis) a suitable model to describe fundamental features of biological membranes? What may be missing?

**DOI:** 10.3389/fpls.2013.00457

**Published:** 2013-11-13

**Authors:** Luis A. Bagatolli, Ole G. Mouritsen

**Affiliations:** ^1^Center for Biomembrane Physics (MEMPHYS), University of Southern DenmarkOdense, Denmark; ^2^Membrane Biophysics and Biophotonics group, Department of Biochemistry and Molecular Biology, University of Southern DenmarkOdense, Denmark; ^3^Department of Physics, Chemistry, and Pharmacy, University of Southern DenmarkOdense, Denmark

**Keywords:** raft hypothesis, fluid mosaic model, membrane lateral pressure profile, membrane compositional fluctuations, membrane curvature, membrane domains, membrane lateral organization

## Abstract

The structure, dynamics, and stability of lipid bilayers are controlled by thermodynamic forces, leading to overall tensionless membranes with a distinct lateral organization and a conspicuous lateral pressure profile. Bilayers are also subject to built-in curvature-stress instabilities that may be released locally or globally in terms of morphological changes leading to the formation of non-lamellar and curved structures. A key controller of the bilayer’s propensity to form curved structures is the average molecular shape of the different lipid molecules. Via the curvature stress, molecular shape mediates a coupling to membrane-protein function and provides a set of physical mechanisms for formation of lipid domains and laterally differentiated regions in the plane of the membrane. Unfortunately, these relevant physical features of membranes are often ignored in the most popular models for biological membranes. Results from a number of experimental and theoretical studies emphasize the significance of these fundamental physical properties and call for a refinement of the fluid mosaic model (and the accompanying raft hypothesis).

## BRIEF HISTORICAL OVERVIEW

Current views on structural and dynamical aspects of biological membranes have been profoundly influenced and to some extent biased by the fluid mosaic model, proposed by Singer and Nicolson (1972). This model supports the idea of lipids forming a more or less randomly organized fluid, flat, bi-dimensional matrix in which proteins perform their distinct functions. Although lipid-mediated lateral heterogeneity in membranes was concurrently described during the 1970s, this feature was not considered in the nascent Singer and Nicolson model.

Early proof that lipids could laterally segregate forming physically distinct “domains” in model membrane systems was reported in the 1970s (Phillips et al., 1970; Shimshick and McConnell, 1973; Grant et al., 1974; Lentz et al., 1976; Schmidt et al., 1977). Along with these observations, it was proposed that lipid compositional heterogeneity may play a role in the modulation of relevant physical properties of natural membranes. Lipid lateral segregation, which might arise under particular environmental plausibly found in physiological states, would be one of these (Gebhardt et al., 1977; Schmidt et al., 1977). Furthermore, membrane regions induced by lipid-protein interactions were proposed as a physical basis for membrane-mediated processes (Marcelja, 1976; Mouritsen and Bloom, 1984; Sackmann, 1984). These and other questions and theoretical possibilities were addressed by various researchers on several occasions (Träuble, 1976; Op den Kamp, 1979; Thompson and Tillack, 1985; Vaz and Almeida, 1993).

To account for lipid-mediated lateral heterogeneity alternative models of biological membranes have been proposed. For example, the “plate model,” introduced by Jain and White (1977), proposed that separation of ordered regions from disordered (fluid) regions occurs in biological membranes as a natural consequence of specific intermolecular interactions and lattice deformation. At around that time, Israelachvili proposed another model to account for the need of membrane proteins and lipids to adjust to each other (Israelachvili, 1977). This insight provided the conceptual framework for “the mattress model” proposed by Mouritsen and Bloom (1984) which suggests that, in membranes, lipids, and proteins exhibit interactions associated with a positive Gibbs energy caused by a hydrophobic matching condition that can lead to elastic distortions of the membrane matrix. This type of phenomenon in turns gives rise to interfacial tension between lipid and proteins, resulting in clustering of specific lipid molecules around a protein or lipid-mediated protein–protein interactions (due to capillary forces). In addition, a model accounting for the importance of the cytoskeleton and the glycocalyx on membrane organization was developed by Sackmann (1995)^1^. Regrettably, many of the important physical mechanism highlighted by these models are generally ignored when membrane-related phenomena are addressed (e.g., transport processes, action of second messengers), and the general outlook introduced by the fluid mosaic model still prevails (Bagatolli et al., 2010; Bagatolli, 2012).

A proposal regarding the role of lipid heterogeneity came along with the “raft hypothesis,” which has its origin in observations reported by Simons and van Meer (1988). These authors envisaged the formation of lipid domains as an early event in the sorting process in the plasma membrane of epithelial cells. This hypothesis was subsequently generalized, proposing the existence of microdomains (“rafts”) enriched in sphingolipids and cholesterol. These domains were surmised to be functionally associated with specific proteins involved in intracellular lipid traffic and cell signaling (Simons and Ikonen, 1997). The idea that these rafts, by being enriched in cholesterol, should have special physical properties arose from original observations in model membranes reported by Ipsen et al. (1987), showing that under particular conditions cholesterol generates the coexistence of liquid-disordered (*l*_ d_) and liquid-ordered (*l*_ o_) lamellar phases. The liquid-ordered phase combines free rotational and translational diffusion of lipids (as found in the L_ d_ phase) with a low proportion of *gauche* rotamers in the hydrocarbon chains (i.e., high order rather than low order), as is usually found in the solid ordered (*s*_ o_, or gel) phase (Ipsen et al., 1987). Since 1997, the raft hypothesis has become very popular among researchers in the biosciences, spawning thousands of projects and publications in multiple areas of cell biology, biochemistry, and biophysics. However, accurate definitions of the physical phenomena that would underlie the raft hypothesis are still lacking, a fact that has resulted in numerous reformulations over the last few years. One of the latest definitions states that rafts are “⋯fluctuating nano-scale assemblies of sphingolipid, cholesterol, and proteins that can be stabilized to coalesce, forming platforms that function in membrane signaling and trafficking” (Lingwood and Simons, 2010). In this definition, “rafts” are claimed to exist in an “ordered phase” (defined as a “raft phase”) that “⋯is not similar to the liquid-ordered phase observed in model membrane systems.” The term *phase* (appropriated from systems at thermodynamic equilibrium) is used in the context of cellular membranes, somehow overlooking that local equilibrium conditions need to hold first. It remains to be established whether membranes are best described as being near local equilibrium at some time scale (thus allowing *phase separation*), or whether they can be more appropriately perceived as metastable regions caused by fluctuations originating from non-equilibrium conditions. Perhaps one of the more questionable aspects of the raft hypothesis was its original operational definition, which was based on detergent extraction methods. Using detergent-extraction techniques is influenced by the way protein chemists work, isolating specific membrane proteins from biological material. However, membranes are self-assembled macromolecular structures in which a range of different molecular species organizes due to weak physical and thermally renormalized forces. Seen from this point of view, adding detergents to membranes is the last thing you would do to study lateral organization. Even though it has been shown that detergents impinges a completely different structural and dynamical features to membranes (Heerklotz, 2002; Sot et al., 2006), the identification of rafts based on various detergent extraction methods is still loosely accepted today. At this stage, however, the fact that detergents do not isolate preexisting membrane domains is more widely recognized (Lingwood and Simons, 2010). Last but not least, conclusive experimental evidence about the existence of rafts in the plasma membrane remains elusive.

## RELEVANT PHYSICAL PROPERTIES OF MEMBRANES

**FIGURE 1:**
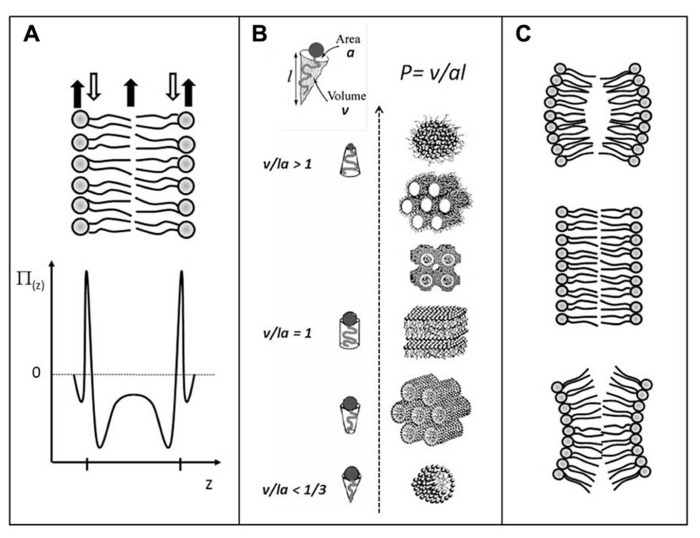
**Schematic illustrations of:** **(A)** the lateral pressure profile, p(z), of a lipid bilayer, revealing regions of expansive (positive) pressures and regions of large tensile (negative) pressures; **(B)** lamellar and non-lamellar lipid aggregates formed by self-assembly processes in water. The different structures have different senses of curvature and are arranged in accordance with the value of the phenomenological molecular packing parameter *P*; **(C)** Lipid monolayers with positive, zero, and negative (from top to bottom) curvature determined by the shape of the lipid molecules. Stable lipid bilayer (center) formed by two opposing lipid monolayers. If the monolayers were not constrained by being in the bilayer, they may curve as shown at the top and the bottom illustrations. In the latest cases, the stable bilayer would suffer from a built-in curvature stress. Adapted from Mouritsen (2011a) with permission.

The structure, dynamics, and stability of lipid bilayers are controlled by thermodynamic forces, leading to overall tensionless membranes with a distinct lateral organization and a conspicuous lateral pressure profile (reviewed in Bagatolli et al., 2010; Mouritsen, 2011a,b). The transverse structure is a noticeable feature of a lipid bilayer, and is far from that of an isotropic fluid slab of hydrocarbons. Bilayers display a distinct lateral stress- or pressure profile (Brown, 1994; Cantor, 1997, 1999a,b; Marsh, 2007) as illustrated in **Figure 1A**. The physics behind this profile is based on simple mechanics. In mechanical equilibrium in the tensionless state, the integral of the difference between the normal pressure and the lateral pressure, p_ N_(z)–p_ L_(z), has to become zero. However, the variation of the lateral pressure across the 5nm thick membrane goes from positive, expansive pressures in the head group region, over regions of negative, tensile pressures in the interfacial regions, to expansive, positive pressures in the acyl-chain region, as illustrated in **Figure 1A**. These variations can easily amount to the equivalent of hundreds of atmospheres pressure. It is this very stressful environment integral membrane proteins have to come to terms with. The lateral pressure profile has recently been computed in 3D (in contract to the initial 1D) and used to determine the effect of the 3D transmembrane pressure distribution on membrane protein activation (Samuli Ollila et al., 2011).

Bilayers are also subject to built-in curvature-stress instabilities that can be locally or globally released in terms of morphological changes (Mouritsen, 2011a,b, 2013). A crucial regulator of the bilayer propensity for forming curved structures is the lipid average molecular shape. It is possible to describe lipid phase behavior via a simple geometric property of the lipid molecule, the so-called Israelachvili–Mitchell–Ninham packing parameter, *P = v/al*, where *v* is the molecular volume, *a* is the cross-sectional area of the head group, and *l* is the length of the molecule (Israelachvili, 1992), cf. **Figure 1B**. Of course a lipid molecule in a dynamic lipid aggregate cannot be assigned a shape as such, and the geometric parameters *v*, *a*, and *l* should therefore be considered as average molecular properties. Still, the value of *P* turns out to be surprisingly useful in predicting the structure of a lipid aggregate. For instance, if the lipid composition in the two leaflets of a thermodynamically stable bilayer changes (e.g., upon lipase action or incorporation of other lipids), and these new lipids have values of *P* different from unity, the bilayer (via the lateral pressure profile) will suffer from a built-in curvature stress. Such monolayers would curve (**Figure 1C**) if they were allowed to do so and not being confined to constitute a stable bilayer, leading to the formation of non-lamellar and curved structures (**Figure 1B**). Via the curvature stress, molecular shape mediates also a coupling to membrane-protein function and provides a set of physical mechanisms for formation of lipid domains and laterally differentiated regions in the plane of the membrane (Mouritsen, 2013).

## ARE THE “FLUID MOSAIC” AND THE ACCOMPANYING RAFT HYPOTHESIS THEN SUITABLE MODELS TO DESCRIBE FUNDAMENTAL FEATURES OF BIOLOGICAL MEMBRANES?

It has been suggested that the fluid mosaic model of membranes has been successful because it does not bias the researcher too strongly, allowing for broad interpretations of new experimental data and novel theoretical concepts (Mouritsen and Andersen, 1998; Bagatolli et al., 2010). This suggestion can somehow be extended to the raft hypothesis. For example, “rafts” have been variously referred to as *constitutive structural elements* of cellular membranes (disregarding important dynamical aspects of membranes), proposed to exist in almost all biological membranes (overlooking their rich compositional diversity), and structured in some sort of liquid-ordered phase (although studies demonstrating the occurrence of local equilibrium conditions are very scarce). Moreover, the assertion of a liquid-ordered structure is seldom verified directly but only indirectly by pointing to the high local concentration of cholesterol. In fact the determination of a liquid-ordered structure has turned out to be an elusive problem even in simple model membranes and only recently has some hard evidence been established in model membranes by combining scattering data and model simulations (Rheinstädter and Mouritsen, 2013). One of the most recent and credible studies of “rafts” in live cells combine fluorescence correlation spectroscopy (FCS) with stimulated emission depletion microscopy (STED; Eggeling et al., 2009). This study suggested the existence of cholesterol concentration dependent domains of sizes around 20nm, where plasma membrane proteins dwell for periods of 10–20ms. The models used for data analysis have, however, challenged because they rely on the assumption of locally “flat” surfaces (supported by the fluid mosaic model) and ignore the already documented complex topography of the plasma membrane (Adler et al., 2010). One way or another, it is clear that the raft hypothesis extends the mosaic nature of the membrane proposed by Singer and Nicolson to include now functionally important distinct *fluid *domains, selective in terms of *both *protein and lipid components. Notice that the generic view of the fluid mosaic model prevails again and no reference is made to relevant membrane physical features such as the transbilayer structure (and the associated lateral pressure profile; Cantor, 1997), curvature stress (Miao et al., 1994), instabilities toward non-lamellar symmetries (Seddon and Templer, 1995), coupling between internal membrane structure or hydrophobic matching (Mouritsen and Bloom, 1984), and intrinsic membrane permeability near phase transitions (Heimburg, 2010). Thus incorporation of other, more realistic, models, or modifications of the most popular ones are urgently required to interpret membrane related phenomena.

## NEW CHALLENGES AND FUTURE PERSPECTIVES

**FIGURE 2:**
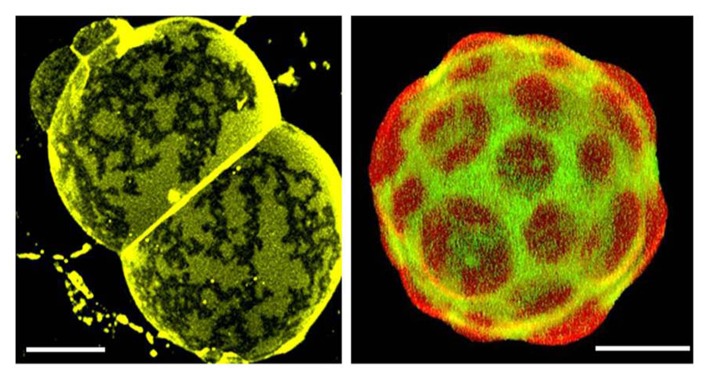
**Confocal fluorescence images of natural membranes showing micrometer-sized domains.** Left: skin stratum corneum lipids membranes from human. This specialized membrane contains 11 different ceramides, cholesterol, and long chain (C_24_–C_26_) fatty acids in a ~1:0.9:0.4 mol ratio, and displays coexistence of two gel-like phases (Plasencia et al., 2007). The membrane is labeled with DiIC_18_, *T* = 32°C (which represent skin physiological temperature. Right: pulmonary surfactant membranes from pig. This specialized membrane is mainly composed of phospholipids and small amounts of specifically associated proteins (SP-B and SP-C). Among the phospholipids, significant amounts of dipalmitoylphosphatidylcholine (DPPC) and phosphatidylglycerol are present, both of which are unusual species in most animal membranes. Mono-unsaturated phosphatidylcholines (PC), phosphatidylinositol, and neutral lipids including cholesterol are also present in varying proportions (Bernardino de la Serna et al., 2004). This natural membrane is labeled with DiIC_18_ (red) and Bodipy-PC (green) and is displaying coexistence of lo and ld-like phases, *T* = 37°C. Scale bars are 10μm.

Are there examples from naturally occurring membranes displaying micrometer-sized domains as observed in model membrane systems? Yes, in very specialized membranes such as lung surfactant and skin stratum corneum, where lipids are the principal components, membrane-cytoskeleton anchorage is lacking, and local equilibrium conditions are likely attainable (Bernardino de la Serna et al., 2004; Plasencia et al., 2007), cf. **Figure 2**. Other examples have been reported, such as platelets upon activation (Gousset et al., 2002), macrophages (Gaus et al., 2003), T-cells (Klammt and Lillemeier, 2012), yeast (Spira et al., 2012), red blood cells (Sanchez et al., 2012; D’Auria et al., 2013), fibroblasts (Frisz et al., 2013) although it is not yet clear whether these observations are controlled by the same mechanisms. The message here is that generalizations can be perilous, and it is probably a good idea to pay attention to the compositional diversity of different membranes, including the way that processes evolve (local equilibrium vs. non equilibrium conditions).

Since conclusive experimental evidence about the existence of domains in live cell plasma membranes remains elusive, fluctuations observed at compositions near the critical point, reported from phase diagrams of ternary mixtures containing cholesterol (Veatch et al., 2007; Idema et al., 2009), have been considered as a potential physical basis to infer the presence of fluctuating nanoscale assemblies in plasma membranes (or rafts). This equilibrium phenomenon is claimed to be relevant to membrane function (Veatch et al., 2008). As mentioned previously (Bagatolli et al., 2010), critical-point phenomena are singular in nature and hence it is unlikely that they *per se* play a role in biological regulation. For example, minuscule mistuning near a critical point may lead dramatic changes in membrane structure and dynamics. It is more likely that a related phenomenon associated with non-equilibrium critical behavior, or self-organized critical behavior, which is robust and needs no tuning, may play a role in biology (Jensen, 1998). Understanding these kinds of processes will prove very challenging, particularly considering that the biophysics of membrane organization under non-equilibrium conditions is in its infancy (Sabra and Mouritsen, 1998; Girard et al., 2005; Turner et al., 2005; Foret and Sens, 2008; Fan et al., 2010; Bouvrais et al., 2012). In order to understand how membrane heterogeneity becomes controlled by the non-equilibrium state of the lipid matrix, it is vital to explore new experimental models and theory-based approaches (Bagatolli et al., 2010; Bagatolli, 2012). For example, *active membrane systems* subject to transport, signaling, and enzymatic processes should be experimentally designed and studied (Bouvrais et al., 2012). Last, but not least, it is worth mentioning that the behavior of biological systems (including membrane related processes) is generally viewed in terms of mass-action kinetics. However, natural systems exist far beyond the dilute concentration limit; consist of molecularly crowded environments with variable water activity and a collection of (small) sizes. The impact of these conditions on membrane structure and dynamics is still obscure and waiting to be elucidated.

## Conflict of Interest Statement

The authors declare that the research was conducted in the absence of any commercial or financial relationships that could be construed as a potential conflict of interest.

## Footnotes

1 Space limitations imposed by the journal for a “minireview article” prevent us to discuss in detail the possibility that cortical actin and its associated proteins could compartmentalize the plasma membrane into distinct domains. Notice however that other contribution in this special issue deals with this topic
